# Support workers knowledge, skills and education relating to dementia – a national survey

**DOI:** 10.3310/nihropenres.13671.2

**Published:** 2024-11-27

**Authors:** Abigail J Hall, Richard Griffin, Fay Manning, Victoria A Goodwin

**Affiliations:** 1 Public Health and Sports Science Department, University of Exeter, Exeter, England, EX1 2LU, UK; 2 Kings Business School, Kings College London, London, UK; 3 Department of Health and Care Professions, University of Exeter, Exeter, England, UK

**Keywords:** Dementia, support workers, healthcare assistants, training, knowledge, attitudes, NHS.

## Abstract

**Background:**

Dementia affects over 55 million people globally, projected to double by 2050. In the UK, non-registered staff, including healthcare assistants (HCAs) and clinical support workers, provide a significant portion of dementia care, yet receive limited training. This study explores the knowledge, training, and attitudes of support workers towards dementia.

**Methods:**

A cross-sectional web-based survey was conducted from February 1 to April 1, 2024, targeting support workers in England. The survey included demographic information, dementia knowledge (using the Dementia Knowledge Assessment Scale, DKAS), and attitudes (using the Dementia Attitudes Scale, DAS). Data were analysed using descriptive statistics, ANOVA, t-tests, and Pearson’s correlation.

**Results:**

One hundred and nine support workers responded, predominantly female (90%) and white British (76.4%), from various NHS settings and occupational groups. The majority (79.8%) had received dementia training, primarily from their organisations. Challenges included managing behavioural and psychological symptoms and communication difficulties. There was no significant correlation between years of experience and dementia knowledge (r = -0.019) or attitudes (r = -0.057). However, higher occupational grades were associated with greater dementia knowledge (p = <0.01). A moderate positive correlation was found between dementia knowledge and attitudes (r = 0.35, p = <0.01).

Despite high levels of knowledge, support workers often feel inadequately prepared to provide optimal dementia care, indicating a need for more comprehensive training. Challenges in communication and managing symptoms highlight areas for improvement. The study suggests that better training can improve both knowledge and attitudes, enhancing care quality for people living with dementia.

**Conclusion:**

Support workers play a crucial role in dementia care but require more robust training programs to meet the growing demands. Enhanced training can improve their knowledge and attitudes, leading to better care outcomes for people with dementia.

## Plain Language Summary

Dementia is a condition that affects millions of people worldwide, with numbers expected to double by 2050. In the UK, many people with dementia are cared for by non-registered staff like healthcare assistants (HCAs) and clinical support workers. These workers provide a lot of direct care but often receive very little training. This study looks at what support workers know about dementia, the training they have received, and their attitudes towards people living with dementia.

We conducted an online survey from February to April 2024, asking support workers across England about their experience, training, and attitudes towards dementia.

We found that how long someone had worked did not significantly affect their knowledge or attitudes about dementia. However, those in higher job positions tended to know more about dementia. There was also a positive link between having more knowledge about dementia and having better attitudes towards people with dementia.

## Background

It is estimated that over 55 million people were living with dementia worldwide in 2020 and this number is projected to approximately double every twenty years, reaching 78 million in 2030 and 139 million in 2050 ^1^. With cognitive decline, there is often an associated physical decline ^2^ and thus an increase in healthcare demands. As it is estimated that 60% of face-to-face patient care in the NHS is provided by non-registered staff ^3^, it can be presumed that a large proportion of people living with dementia (PLwD) will receive face-to-face care from a non-registered professional. There are almost 400,000 healthcare assistants (HCAs) and clinical support workers in the UK who work alongside nurses and other health professionals ^4^ and despite providing the majority of face-to-face care, evidence suggests that non-registered staff only receive 5% of the NHS’ training budget ^3^.

The Dementia Training Standards Framework (DTSF) ^5^, provides a structured guide for dementia training across health and social care sectors. The framework outlines key competencies at various levels, recommending that all staff, including support workers, complete level 1 (awareness) and level 2 (intermediate) training to ensure a foundational understanding of dementia care. Although the DTSF is widely recognised for setting baseline competencies in dementia care across health and social care roles, but specific evaluations of its impact on support workers, especially in terms of long-term outcomes, are limited.

There is little research investigating the effectiveness of support workers working with PLwD ^6^, nor is there any evidence about the knowledge of support workers about dementia. One systematic review ^7^ suggested a positive effect of support workers on the quality of life for PLwD and the reduction in carer burden – however, it also highlighted the lack of high-quality evidence. The NHS Long Term Plan ^8^ and the NHS Long Term Workforce Plan ^9^ recognise the importance of support workers to deliver effective and safe NHS services. The Long Term Workforce Plan particularly emphasises the importance of promoting routes to professional qualifications for support workers to expand their scope of practice. However, the availability and uptake of training for support workers relating to dementia remains unclear despite the increasing demand for healthcare professionals to be able to effectively care for PLwD. Thus, this study aimed to explore the knowledge that support workers have relating to dementia and the access to training and support that they have received. It also sought to understand the thoughts and attitudes of support workers toward dementia and whether there is any correlation between knowledge and attitudes toward people living with dementia.

## Methods

A cross-sectional web-based survey was undertaken using JISC Online Surveys, which is subscribed to by the University of Exeter. The survey was open from 1 ^st^ February 2024 to 1 ^st^ April 2024 and employed a convenience sampling strategy.

An introduction and a link to the participant information sheet including the inclusion criteria was provided on the first page of the survey. There was also information provided about GDPR. To complete the survey, individuals were asked to confirm that they were a support worker working in England and regularly meet patients/clients who are living with dementia.

Respondents consented to participate by clicking the final submit button once the survey was completed. Survey responses were anonymous unless contact details were provided for study updates or other research opportunities. These details were stored securely and separately from the survey responses. Although it was anticipated that there would be little identifying data given by respondents, there was the option for free text where identifying data could be entered, therefore, all data were checked and any identifiable data was removed/anonymised to prevent identification. This anonymisation was completed before the analysis.

### Inclusion criteria

- Support worker with current or previous experience of working in the NHS
- Regularly work with older adults who may have dementia or cognitive impairment

### Recruitment & consent

A targeted social media campaign using “X” (formerly Twitter) was used to advertise the survey, specifically aiming to reach Allied Health Professional (AHP) support workers and nursing support workers. Relevant professional bodies were contacted and asked to disseminate the survey link to their associate (non-registered) members, including those in nursing roles. Additionally, an email was sent to regional Allied Health Profession leads, who were asked to share the survey with both AHP and nursing support worker networks within their regions and organisations. The survey included demographic information relating to the sampling strategy (gender, ethnicity, professional background, location of work, years of experience etc) and to ensure maximum variation, several waves of promotion of the study occurred focusing on groups or characteristics that were underrepresented. This included support workers who work alongside a particular profession that had received fewer responses.

Participants self-identified as support workers within the NHS, including those in Bands 2 to 6. Although Band 5 and 6 roles typically carry more senior responsibilities, we included these individuals as their primary role was reported as a support worker. This inclusion allows for a broader understanding of the knowledge and attitudes across the spectrum of support workers, although it may reflect a wider scope of experience.

### Data collection

JISC Online Surveys™ (JISC, https://www.jisc.ac.uk/online-surveys) was used to collect responses to the survey. The survey was piloted with a small number of support workers (n=10) before roll out. These were selected from a support worker advisory group we have developed to identify any issues with understanding, formatting, and time to complete. It was estimated that the survey would take 15 minutes to complete which was explained on the Participant Information Sheet (PIS). Following the pilot, no revisions were needed to be made to the survey and their data were included in the analysis. There were no incentives offered to take part in the survey. The survey was open for two months following approval to begin data collection. A total of 58 questions were asked, over three separate pages. JISC allows the participant to move back to a previous page should they have wished to review their answers before submitting the survey.

The survey included questions on professional background, qualifications, and role. We then sought to understand their knowledge about dementia and any training they have received to support them in their role. Further to this, we aimed to understand their attitude towards dementia. The survey included both open and closed questions. Closed questions involved a tick box, multiple choice answers, or a Likert rating scale. Respondents were also allowed to answer “other” where the pre-specified options may not have applied. There were also open questions offering the opportunity for the respondent to offer free text answers. All questions were optional to allow respondents to feel free to answer the questions they felt comfortable with.

T used the Dementia Knowledge Assessment Scale (DKAS) ^10^ and the Dementia Attitudes Scale ^11^ were used. The DKAS is a 25-item measure of understanding dementia that is comprised of verifiably true and false statements about dementia, with higher scores suggesting greater knowledge relating to dementia. The scale has been validated with large Australian and international samples (n > 3,500) of health professionals (nurses, aged care workers, physiotherapists, occupational therapists, health educators, and students) and lay respondents ^10^. It comprises statements about dementia that are factually correct or incorrect, which were developed following a Delphi study with dementia experts. An adapted version of the Dementia Knowledge Assessment Scale (DKAS) was used, developed by Annear *et al*. ^10^, which offered the options of ‘True’, ‘False’, or ‘Don’t know’ to ensure ease of completion. Additionally, we explored the attitudes of the support worker to PLwD using the Dementia Attitude Scale (DAS), developed by O'Connor and McFadden ^11^). The seven-point scale includes twenty items ranging from strongly disagree to strongly agree scoring between 20 and 140, with higher scores suggestion a better attitude to dementia.

### Patient and Public Involvement

The basis for this study was informed by patients and the public, who reported that often clinicians did not seem to have a good understanding about dementia, nor did they always understand how best to approach and support them or their relatives.

## Analysis

Before analysis, all data were checked and cleaned. Two responses were excluded as the participant noted that they were not working as a support worker. Any duplicate entries were removed if evident. Descriptive statistics (mean, standard deviation, frequencies, and percentages) were calculated for closed questions using the analysis function provided by JISC.

For the validated questionnaires, we compared continuous data (e.g., mean DKAS and DAS scores) with categorical variables (e.g., years of experience) using parametric tests, such as Analysis of Variance (ANOVA). We also sought to explore whether there was any correlation between knowledge and attitude towards dementia using Pearson’s correlation. For the purpose of statistical analysis, the "occupational grade" variable was numerically coded to enable comparisons. Each NHS band (e.g., Bands 2, 3, 4) was assigned a corresponding numeric value to facilitate statistical testing. For example, comparisons were made between Bands 2 and 4 by using these coded values to compute t-tests and correlations, allowing us to examine potential differences in knowledge and attitudes between these groups. Before comparing variables, the Kolmogorov–Smirnov test was used to determine the normality of the distribution to guide the statistical analysis. Independent t-tests were used to compare between group differences.

For questions involving free text responses, answers were collated and grouped into themes. Data were downloaded into Excel, each row representing individual respondents and each column the research questions. Survey results were reported according to the Checklist for Reporting Results of Internet E-surveys (CHERRIES) ^12^.

### Ethical approval and consent

Ethical approval was granted on the 11 ^th^ January 2024 from the Kings College London – reference MRA-23/24-41002. Informed consent was obtained electronically from all participants. All procedures, including the informed consent process, were conducted in accordance with the ethical standards of the responsible committee on human experimentation (institutional and national) and with the Helsinki Declaration of 1975, as revised in 2000.

### Data protection and participant confidentiality

All data were stored on Kings College London’s One-Drive. Only the primary researchers had access to this. Storing all data on this central server prevented the need to share data between the University of Exeter and Kings College London.

JISC online was used to collect responses to the survey. JISC Online Surveys (formerly Bristol) online survey tool ( https://www.onlinesurveys.ac.uk/). The JISC data is secure and strict information security standards are followed (ISO27001) and data is processed in compliance with GDPR.

Data will be stored for 5 years after the end of the study.

## Results

One hundred and nine unique respondents completed the survey ( Table 1) representing a variety of professional groups working in a variety of different settings. The support workers were spread over the seven geographical regions as categorised by NHS England.

**Table 1.** Characteristics of participants.

		N (%)
Gender	Male (including transgender men)	7 (6.4)
	Female (including transgender women)	97 (90)
	Non-binary	2 (1.8)
	Other	1 (0.9)
	Prefer not to say	1 (0.9)
Ethnicity	White – British, Irish or any other background	88 (81)
	Mixed – White and Black Caribbean, African Asian or any other background	4 (3.6)
	Asian or Asian British	6 (5.4)
	Black or Black British–Caribbean, African, any other	6 (5.4)
	Chinese	1 (0.9)
	Any other groups	2 (1.8)
	Not stated	1 (0.9)
Location of work (by NHS England region)	East of England	7 (6.4)
	London	7 (6.4)
	Midlands	22 (20)
	North East and Yorkshire	4 (3.6)
	North West	18 (16.4)
	South East	18 (16.4)
	South West	33 (31.8)
Occupational group	Nursing	52 (48)
	Occupational Therapy	20 (18.3)
	Physiotherapy	18 (16.5)
	Other	20 (18.3)
	Speech and language therapy	9 (8.3)
	Radiography	5 (4.6)
	Dietetics	4 (3.7)
	Social care	3 (2.8)
	Pharmacy	1 (0.9)
	Public Health	1 (0.9)
	Healthcare science	1 (0.9)
	Prefer not to say	2 (1.8)
NHS Grade	2	24 (22)
	3	34 (31)
	4	3 (32.1)
	5	8 (7.3)
	6	4 (3.7)
	Other/unsure	3 (2.7)
Setting of Work	Acute hospital	55 (50.5)
	Rehabilitation hospital	13 (11.9)
	Community Hospital	11 (10.1)
	Community team	28 (25.7)
	GP Surgery	2 (1.8)
	Other	11 (10.1)
Length of NHS service	< 1 year	10 (9.5)
	1 – 5 years	30 (28)
	6-10 years	21 (20)
	11 – 15 years	8 (7.6)
	16 – 20 years	9 (8.6)
	20+ years	25 (23.8)

### Challenges

Participants were asked to identify the main challenges they faced when working with individuals with dementia. While the challenges were diverse and multifaceted, several common themes emerged from their responses. Of the 109 responses, 90 participants (83%) reported challenges supporting PLwD.

The most frequently reported challenge (32% of participants) was behavioural and psychological symptoms that make it challenging to care for this group of people, including physical and verbal aggression as well as fluctuating levels of confusion.

*Some of the patients can be very aggressive and more recently the patients we get in our ward have been the worst we’ve ever had and with no restraints we are now often getting injured also they can be verbally aggressive and swear a lot not all but when there’s a ward of 21 dementia patients and you have around 8 both male and female then the work is very hard and challenging*

Communication issues were also significant, with 29% of support workers struggling to communicate with the person with dementia and 11% finding it difficult to communicate with the person's family or relatives, either due to difficulty reaching them or supporting them to understand the situation. Additionally, 7% of participants reported that getting individuals with dementia to follow instructions hindered their ability to perform their roles effectively.

*Communication can be challenging when working with people with dementia. If their dementia is more advanced, it can be difficult to obtain coherent responses to the questions we ask. I often rely on a carer/relative being present at home visits as they are able to provide essential information about their loved one.*

A lack of time and resources was noted by 13% of participants, which directly impacted their ability to get to know the person and understand their preferences. Other challenges included addressing physical needs, such as meeting nutritional requirements (4/92), ensuring physical safety (3/92), and providing adequate care. Furthermore, 13% of participants reported difficulties in accessing social care or obtaining support for the person with dementia.

### Training

Participants were asked if they had received any specific training relating to dementia. Eighty-seven (79.8%) respondents reported that they had received specific training and 54 (49.1%) reported that they had completed mandatory training relating to dementia. While most participants had received some training, many (78%) felt that further training would be useful to effectively manage PLwD. Several participants felt that dementia training should be mandatory.

*I think Dementia training should be mandatory for all health and social care professionals. This should include how to support the person, and their family, dealing with behaviours that may challenge you*

Of those who had received specific training, the majority had received training from their organisation (or previous organisations) (89%) with only a small number (11%) having been able to attend any external training. External training included diplomas, stand-alone courses at Higher Education Institutes or one person reported having undertaken a Master's degree in dementia. Other specialist courses were rarely undertaken by our participants – Teepa Snow (1%) and the “Dementia Bus” (6%). Sixty-seven percent of participants who responded reported that they had received training in other mental health conditions such as Autism, Schizophrenia, depression, and anxiety. Participants reported that further training would be useful, particularly around improving communication skills with PLwD and simulation to allow them to get a better understanding of how it feels to live with dementia.

*Yes, simulation would be nice. Most people do not understand how the person living with dementia communicate their needs. I think this should be where focus on training should lie, on interpreting challenging behaviours instead of just generalising that challenging behaviour is a normal part of the disease.*

Many participants reported that without the availability of in-depth training related to dementia, much of their knowledge was self-taught through many years of experience working with this population.

### Experience

We sought to evaluate whether the level of experience of the support worker affected the knowledge or attitudes towards dementia.

***Years of experience and attitude towards people with dementia***

The analysis revealed a Pearson correlation coefficient of -0.057 between years of experience and DAS score. This value indicates a very weak negative correlation, suggesting that there is no meaningful relationship between the years of experience and the DAS score in this sample.

For NHS workers with 1–5 years of experience, the mean DAS score was 107.2 (95% CI 102.30 - 112.10). In contrast, those with 20+ years of experience had a mean DAS score of 105.65 (95% CI 97.85 - 113.45). The independent two-sample t-test yielded a t-statistic of 0.36 and a p-value of 0.719, indicating no statistically significant difference between the two groups (p > 0.05).

These results suggest that the attitudes towards dementia, as measured by the DAS, do not significantly differ between NHS workers with less than 5 years of experience and those with over 20 years of experience. These results suggest that, among those who remain in support worker roles, the length of time working within the NHS does not significantly influence attitudes toward dementia as measured by the DAS score. However, it is also possible that changes in attitudes could influence career trajectory, with some individuals who develop different perspectives or attitudes over time potentially leaving support worker roles. This potential attrition effect may mean that our sample reflects the attitudes of those who remain in the role, rather than capturing attitude changes over time within the broader population.

***Years of experience and knowledge of dementia***

The analysis revealed a Pearson correlation coefficient of -0.019 between years of experience and DKAT score. This value indicates an extremely weak negative correlation, suggesting that there is no significant relationship between the years of experience and the DKAT score in this sample.

For NHS workers with 1-5 years of experience, the mean DKAT score was 38.0, ( 95% CI 35.94 - 40.06). In contrast, those with 20+ years of experience had a mean DKAT score of 36.96 (95% CI - 34.30 to 39.61). The independent two-sample t-test yielded a t-statistic of 0.65 and a p-value of 0.519, indicating no statistically significant difference between the two groups (p > 0.05).

These results suggest that the level of dementia knowledge, as measured by the DKAT, does not significantly differ between NHS workers with less than 5 years of experience and those with over 20 years of experience.

The extremely weak negative correlation observed between years of experience and DKAT score, much like the DAS score, indicates that years of experience within the NHS do not significantly influence dementia-related knowledge. The weak negative correlation observed between years of experience and DAS score indicates that other factors might play a more significant role in shaping attitudes towards dementia among support workers.

### Occupational grade

We further sought to determine whether there was any difference in knowledge and attitudes between occupational grades

***Occupational grade and knowledge of dementia***

The analysis revealed a significant difference in DKAS scores between Band 2 and Band 4 workers, with Band 4 workers scoring higher on average (mean DKAS score for Band 2 = 34.92, 95% CI: 32.46–37.37; mean DKAS score for Band 4 = 39.18, 95% CI: 37.11–41.25; t = -2.72, p < 0.01). However, given the weak, statistically non-significant overall correlation between occupational grade and dementia knowledge (Pearson correlation coefficient = 0.076, p = 0.452), this finding may reflect the relatively early career stage of Band 2 workers, who likely have less experience and fewer training opportunities specific to dementia. This difference, therefore, may not suggest that higher grades inherently possess greater dementia knowledge but rather that those in Band 2 are often in starter roles where dementia-specific knowledge is less developed.

***Occupational grade and attitude towards dementia***

To explore the relationship between occupational grades (NHS bands) and attitudes towards dementia, as measured by the DAS score, a Pearson correlation analysis was performed. The “occupational grade” variable was numerically coded for this analysis.

The analysis revealed a Pearson correlation coefficient of 0.004 between occupational grade and DAS score. This indicates a very weak positive correlation. The p-value for this correlation was found to be 0.967 which indicates that the observed correlation is not statistically significant.

An independent two-sample t-test was conducted to compare the DAS scores between Band 2 and Band 4. The mean DAS score for Band 2 was 101.67 (95% CI 92.99 - 110.35), while the mean score for Band 4 was 107.59 (95% CI 102.06 - 113.12). The independent two-sample t-test yielded a t-statistic of -1.24 and a p-value of 0.221. These results indicate that there is no statistically significant difference in DAS scores between the two occupational grades (p > 0.05). The results indicate that there is no significant relationship between the occupational grade of NHS employees and their attitudes towards dementia.

### Knowledge of dementia and attitude towards dementia

A Pearson correlation analysis was conducted to evaluate the relationship between DAS and DKAT scores. The Pearson correlation coefficient between DAS and DKAT scores was found to be 0.35 (p=<0.01), indicating a moderate positive correlation. This suggests that higher knowledge about dementia is associated with more positive attitudes towards dementia.

## Discussion

This study aimed to explore the knowledge that support workers have relating to dementia and the access to training and support that they have received. It also sought to understand the thoughts and attitudes of support workers toward dementia and whether there is any correlation between their knowledge and attitudes. Our data suggests that while the majority of participants had received training relating to dementia, they did not feel it was sufficient to be able to give patients the optimum care, despite their knowledge generally being high.

The knowledge levels observed in our sample, as measured by the DKAS, appear higher than those reported in previous standardisation studies among support workers ^10^. This may suggest that dementia training programs in England, guided by frameworks like the DTSF, have a positive effect on knowledge levels. Similarly, the attitudes observed in our sample align with positive attitudes reported by O’Connor and McFadden ^11^ in their work with the Dementia Attitudes Scale (DAS), indicating that training may also support compassionate attitudes in dementia care settings.

While our findings indicate gaps in knowledge and confidence, it is possible that some support workers may not have full access to or awareness of the DTSF resources. Therefore, improving accessibility and awareness of these established training pathways may be a cost-effective strategy for enhancing knowledge across support worker roles. Additionally, given that the DTSF and similar training frameworks are designed to provide standardised skills across different care roles, targeted reinforcement of the DTSF competencies could be beneficial, especially if integrated into routine supervision or mentorship programs within the NHS.

To ensure training is both effective and cost-effective, future initiatives might consider integrating simulation-based training and peer support, which have been shown to improve practical skills in dementia care ^13^. Simulation exercises could help support workers experience the challenges faced by individuals with dementia, fostering empathy and improving their ability to respond to challenging behaviours. Peer-led reflective sessions, where workers share experiences and discuss strategies for managing symptoms, could also support practical application of the DTSF competencies without requiring extensive additional resources.

There is evidence to suggest that training programs enhance support workers’ understanding of dementia, leading to better care practices. Approaches such as “Dementia Care Mapping” have been shown to improve caregivers’ skills in recognising and responding to the needs of PLwD ^14^. Many of our participants reported challenges communicating effectively with PLwD. Evidence suggests that training improves communication skills, enabling support workers to interact more effectively. This can lead to improved patient outcomes, such as reduced agitation and improved cooperation with care routines ^15^. Further challenges cited were around the challenges of managing behavioural and psychological symptoms (BPSD) and studies indicate that bespoke training can aid support workers to better manage these symptoms of dementia ^13^.

Our analysis was conducted to explore the relationship between years of experience, occupational banding, and attitudes and knowledge about dementia found no significant relationship between years of experience and attitudes (DAS score) or knowledge (DKAT score) about dementia. Similarly, there was no significant relationship between occupational level and attitudes towards dementia. However, a significant difference in dementia knowledge was observed between different occupational levels, with higher occupational levels showing greater knowledge. These findings suggest that neither years of experience nor occupational grading levels significantly influence attitudes towards dementia, though higher bands may have better dementia knowledge.

Studies indicate that those who receive specialised training in dementia care tend to have more positive attitudes and are better equipped to provide high-quality care ^15^. The World Alzheimer Report 2019 by Alzheimer's Disease International highlights the varying attitudes towards dementia, including those of support workers. The report found significant differences in attitudes, with some caregivers showing high levels of empathy and others expressing stigmatising beliefs about dementia ^16^.

Our data suggested a moderate positive correlation between knowledge and attitudes toward people living with dementia (PLwD). However, it is essential to note that correlation does not imply causation, and the association should not be interpreted as direct evidence that increased knowledge causes improved attitudes. While training may contribute to the knowledge levels observed, our study did not assess the specific sources of knowledge, meaning we cannot confirm that training alone is responsible. This supports other findings which suggest that training programs provide support workers with a better understanding of dementia, including its symptoms, progression, and the needs of those affected ^17^. By addressing common myths and stigma associated with dementia, training may help support workers develop more accurate views of PLwD and strategies to help support them. Educational sessions that highlight the abilities and potential of PLwD, rather than just their limitations, can reduce stigmatising beliefs ^18^.

While our study highlights the role of training in enhancing dementia knowledge and improving attitudes among support workers, we recognise that training alone may not be sufficient to prepare staff for the complexities of dementia care. Existing literature suggests that a supportive organisational environment, regular supervision, and access to mentorship are essential in reinforcing knowledge and sustaining positive attitudes ^15^. Organisational culture that fosters openness, empathy, and collaboration may empower support workers to address challenging behaviours and communication barriers in ways that training alone cannot achieve. Ongoing support from supervisors and peers, along with structured opportunities for reflection and feedback, can be especially effective in helping staff apply learned skills in real-world contexts. This layered approach, integrating formal training with organisational and team-based support, is likely to build resilience and confidence, ultimately improving the quality of dementia care.

## Conclusion

Our findings indicate that while support workers generally possess a high level of knowledge about dementia, many feel inadequately prepared to provide the level of care needed by individuals living with dementia, especially when facing challenges related to communication and managing behavioural symptoms. This suggests a pressing need not just for additional training but for more targeted, accessible, and practical dementia care training tailored to support workers’ unique needs.

The Dementia Training Standards Framework (DTSF) offers a foundational structure, yet current training delivery methods may be insufficient for equipping support workers with the practical skills required in everyday settings. Given that support workers comprise one of the largest workforce groups in the NHS, yet receive a small proportion of the training budget, it is essential for healthcare leaders, policy makers, and NHS Education bodies to consider cost-effective, flexible, and scalable training solutions. Options such as online learning modules, simulation-based training, and blended learning approaches—combining digital training with in-person supervision or peer-led reflective sessions—could be instrumental in enhancing skill development without significantly increasing costs.

To implement these programs effectively, a collaborative approach is required. Stakeholders such as NHS training bodies, healthcare providers, and charitable organisations specialising in dementia care (e.g., Alzheimer’s Society UK) could play critical roles in producing and delivering this training. Funding could be sourced through NHS training budgets or collaborative grants, with a focus on scalable methods that can accommodate the varied schedules and resource constraints of support workers.

Ensuring that support workers are equipped with comprehensive dementia care skills requires strategic investment from NHS leadership and a re-evaluation of budget allocation. By dedicating resources specifically for dementia care training for support workers, the NHS can support this essential workforce group, ultimately improving patient outcomes and the quality of dementia care. We urge policy makers to prioritise this investment, recognising the substantial and direct impact that well-prepared support workers have on the lives of people living with dementia.

## Strengths and limitations

We recognise that the relatively small number of respondents could not be considered representative to the whole population of support workers in England, however, the survey offers insights into the training needs and requirements for our support workforce. However, we were able to sample a wide range of different groups of support workers across a variety of different settings to gain insights into the workforce as a whole.

Our study relied on an online survey platform, which may not have been readily accessible to all support workers. Barriers such as limited digital resources, literacy challenges, and English fluency could have restricted participation among certain groups, potentially skewing the demographics of our sample. Notably, the majority of respondents identified as white British, which may reflect an accessibility limitation that could affect the generalisability of our findings.

To ensure respondents felt at ease and were able to participate fully, all questions were optional. This approach aimed to minimise any potential discomfort or disengagement by allowing respondents the flexibility to answer only those questions they felt comfortable with. Although dementia care is not typically considered a sensitive topic, we adopted this approach to encourage honest responses across a diverse workforce with varying levels of comfort and experience with dementia care. However, this flexibility may have led to some incomplete responses. We acknowledge this as a limitation, as it may have resulted in missing data for certain questions, potentially affecting the comprehensiveness of the dataset.

A small proportion (11%) of participants identified as Band 5 or Band 6, levels which generally entail more senior responsibilities beyond traditional support worker duties. While these participants identified as support workers, it is possible their views and knowledge could be influenced by higher-band responsibilities, which may differ from those in lower-band roles. Future studies might consider eligibility criteria to focus on Bands 2–4, ensuring the sample aligns closely with typical support worker roles.

## Ethical approval and consent

Ethical approval was granted on the 11 ^th^ January 2024 from the Kings College London – reference MRA-23/24-41002 .Informed consent was obtained electronically from all participants. All procedures, including the informed consent process, were conducted in accordance with the ethical standards of the responsible committee on human experimentation (institutional and national) and with the Helsinki Declaration of 1975, as revised in 2000.

## Data Availability

The anonymised dataset is accessible for review. Open Science Framework: Support workers knowledge, skills and education relating to dementia – a national survey DOI: osf.io/rwty7 ^13^ This project contains the following underlying data: Cleansed anonymised data (Excel spreadsheet with anonymised data set) The survey questions are accessible for review. Open Science Framework – DOI: osf.io/rwty7 ^13^ This project contains the following extended data: Survey questions (Word document detailing questions used in the survey) Data are available under the terms of the CCO 1.0 Universal license.

## Software availability statement

The online survey used in this study was conducted using JISC Online Surveys. The survey tool is accessible at [ https://www.jisc.ac.uk/online-surveys] ( https://www.jisc.ac.uk/online-surveys). It is a commercial tool available under a subscription license. Data collected through the survey is stored securely and complies with relevant privacy regulations, including GDPR. Documentation for the survey tool can be found at [ https://www.jisc.ac.uk/online-surveys] ( https://www.jisc.ac.uk/online-surveys). For further inquiries, please contact the JISC support team at help@jisc.ac.uk.
