# Supplementary material for: Support workers knowledge, skills and education relating to dementia – a national survey
Source: NIHR Open Res. 2024 Nov 27;4:54. Originally published 2024 Sep 24. [Version 2] doi: 10.3310/nihropenres.13671.2 (PMC11605170; doi:10.3310/nihropenres.13671.2)
Supplement: Peer review history

# Reviewer report: Reviewer response for version 2

**DOI:** 10.3310/nihropenres.15025.r33851

**Published:** 2025-01-07

**Authors:** Julie Nightingale, Sarah Etty

**Affiliations:** 1 CARe Research Centre, Sheffield Hallam University College of Health Wellbeing and Life Sciences (Ringgold ID: 111995), Sheffield, England, UK

The amendments made have strengthened the article. No further amendments suggested.

Is the work clearly and accurately presented and does it cite the current literature?

Partly

If applicable, is the statistical analysis and its interpretation appropriate?

Yes

Are all the source data underlying the results available to ensure full reproducibility?

Yes

Is the study design appropriate and is the work technically sound?

Yes

Are the conclusions drawn adequately supported by the results?

Partly

Are sufficient details of methods and analysis provided to allow replication by others?

Yes

Reviewer Expertise:

Reviewer 1- Workforce research, support worker research; Reviewer 2 - support worker research, quantitative analysis

We confirm that we have read this submission and believe that we have an appropriate level of expertise to confirm that it is of an acceptable scientific standard.

# Reviewer report: Reviewer response for version 2

**DOI:** 10.3310/nihropenres.15025.r33850

**Published:** 2024-11-28

**Authors:** Jan Oyebode

**Affiliations:** 1 University of Bradford, Bradford, England, UK

I have just re-read through the report and appreciate the changes made by the authors. I think it reads well and is a very useful addition to the literature.

Is the work clearly and accurately presented and does it cite the current literature?

Partly

If applicable, is the statistical analysis and its interpretation appropriate?

Yes

Are all the source data underlying the results available to ensure full reproducibility?

Yes

Is the study design appropriate and is the work technically sound?

Yes

Are the conclusions drawn adequately supported by the results?

Partly

Are sufficient details of methods and analysis provided to allow replication by others?

Yes

Reviewer Expertise:

Dementia care research including young onset dementia, ethnicity and dementia, dementia training and education.

I confirm that I have read this submission and believe that I have an appropriate level of expertise to confirm that it is of an acceptable scientific standard.

# Reviewer report: Reviewer response for version 2

**DOI:** 10.3310/nihropenres.15025.r33221

**Published:** 2024-11-28

**Authors:** Stephen M Quick

**Affiliations:** 1 Allied Health Research, Northern Health (Ringgold ID: 3235), Epping, Victoria, Australia; 2 La Trobe University School of Allied Health Human Services and Sport (Ringgold ID: 110570), Melbourne, Victoria, Australia

Thank you for the opportunity to review this article.

 The paper looks at the incredibly important topic of support worker knowledge and training in dementia care, and presents findings from a national survey.

 In summary, the study is well written overall and addresses an important area of research. Please see below for some more specific information and thoughts as a reader of this article. I note on submitting this review that there have been revisions made in the last 48 hours in relation to feedback from other reviewers, so I will endeavour to not be repetitive in these already addressed areas.

**Abstract**:

*Background section: *Consider revising the discussion on prevalence between the abstract and the beginning of the background for improved coherence. The abstract highlights ‘Dementia affects over 55 million people globally, projected to double by 2050’, whilst the start of the background mentions ‘It is estimated that over 55 million people were living with dementia worldwide in 2020 and this number is projected to approximately double every twenty years, reaching 78 million in 2030 and 139 million in 2050’. There is quite a disparity between 110 million (double 55 million) and the projected 139 million in the background. Perhaps changing the abstract to ‘Dementia affects over 55 million people globally, with projections suggesting this will increase to approximately 139 million by 2050’ or similar may assist in consistency.

*Background section:* Consider referring to UK as the United Kingdom (UK) in the first instance.

*Plain language summary: ‘*We found that how long someone had worked did not significantly affect their knowledge or attitudes about dementia.’  Perhaps ‘….worked **as a support worker** did not significantly affect their….’ may add some clarity here?

**Background:**

*Page 4 Para 1: *Consider referring to the National Health Service (NHS) in the first instance. This may improve context and understanding, particularly for international readers of the research.

*Page 4 Para 1: *Consider if the last sentence of the first paragraph would better fit as the second sentence of the second paragraph. This may allow for a linking sentence at the end of paragraph one highlighting the impact of increased prevalence may have on the NHS into the future, and setting up for the second paragraph regarding support workers.

*Page 4 Para 1: *Is it possible for more of a description of the role of healthcare assistants/support workers in the UK context? These roles have different names and definitions in different countries, so it would be good to even have a brief understanding of the key roles generally played by a support worker in general care, particularly as an international reader.

**Methods**

*Page 4 Para 3: *‘The basis for this study was informed by patients and the public, who reported that often clinicians did not seem to have a good understanding about dementia, nor did they always understand how best to approach and support them or their relatives.’ – is there further supporting evidence or context to this statement? Reads quite anecdotal in the current form.

*Page 4 Para 4: *It may be worth mentioning General Data Protection Regulation (GDPR) on first mention in the paper.

*Page 4 Para 10 (Data Collection): *The authors note that 'Following the pilot, no revisions were needed to be made to the survey.' It would be helpful to understand how this conclusion was reached - what criteria or feedback from the pilot participants indicated that the survey was ready to be sent out in its original form, or was it based solely on understanding, formatting and time to complete?

*Page 5 Para 3:* Were there any duplicate entries, and if so, how were they determined? Was it IP address, responses etc.?

*Page 5 Para 6: *Can you provide more information about the process of collating and grouping into themes? How many authors were involved, what steps comprised the overall process to arrive at these themes, and what were the themes identified?

**Results**

*Page 5 Para 12:* ‘several common themes emerged from their responses’. Although it is not completely clear the analysis method used for the free text data, themes generally do not emerge from the data, with the authors constructing themes through their analysis and understanding of the material. Perhaps something along the lines of ‘…through analysis, we identified several common themes…’ or something similar could be considered.

*Page 5 Para 12: *This section may benefit from a mention of the topics to be discussed in the following paragraphs (ie. Behavioural and psychological symptoms, communication, and lack of time and resources) if it fits with the flow of the results in your opinion.

*Table 1:* The layout of Occupational Group isn’t clear. All rows add to up well over 109 unique respondents, or is each option below ‘other’ then listing the professions that make up ‘other’? In this instance, Nursing (52), OT (20), PT (18) and Other (20) adds up to 110 and not 109. Were participants able to provide multiple responses? More clearly presenting the data in table form (or detailing where people may have been able to select multiple response or did not answer the question) may assist with reading and interpreting this table for the audience.

*Table 1:* NHS Grade – assuming the percentages are correct, this should be 35 NHS Grade 4 support workers? This equals 108 – was this skipped by one participant? Listing the number of respondents (if different to the full sample of 109) may help with interpretation of the table for the reader in some instances.

*General: *Is it possible to add more contexts to the quotes? For example, a quote on a challenge may have more of an impact on a reader if it is coming from a Grade 6 than perhaps a Grade 2, given experiential learning, experience etc. and the support worker still highlighting such challenges.

*Consistency:* The results section would benefit from consistency in reporting either numbers/percentages or n=, or both. For example, lack of time and resources was noted by 13% of participants, however I am not sure if the full 109 participants responded to this question. Including % and n= may help with consistency of reading and interpretation for the reader.

*Consistency*: Likewise with reporting of CI’s and data, there is variation paragraph to paragraph. Some have a space between the ‘(‘ and ‘CI’ (see *years of experience and knowledge of dementia’)*, others have a ‘-‘ between the numbers, whereas others have ‘ranging from’. Consistency in how the information is delivered will assist with the readability of the section.

**Discussion:**

 This is also discussed in the abstract and plain language statement, however I do feel on reading that the phrasing that better training ‘can’ improve both knowledge and attitudes may need to be softened a touch based off these findings alone. From my understanding, cross-sectional correlations cannot prove causation. Therefore I would suggest softening of the language around the results, and this could also lead to potential future directions of the research.

Is the work clearly and accurately presented and does it cite the current literature?

Partly

If applicable, is the statistical analysis and its interpretation appropriate?

Yes

Are all the source data underlying the results available to ensure full reproducibility?

Yes

Is the study design appropriate and is the work technically sound?

Yes

Are the conclusions drawn adequately supported by the results?

Partly

Are sufficient details of methods and analysis provided to allow replication by others?

Yes

Reviewer Expertise:

PhD completed in the area of physiotherapy and the care of people living with dementia.

I confirm that I have read this submission and believe that I have an appropriate level of expertise to confirm that it is of an acceptable scientific standard, however I have significant reservations, as outlined above.

# Reviewer report: Reviewer response for version 1

**DOI:** 10.3310/nihropenres.14844.r33223

**Published:** 2024-11-13

**Authors:** Julie Nightingale, Sarah Etty

**Affiliations:** 1 CARe Research Centre, Sheffield Hallam University College of Health Wellbeing and Life Sciences (Ringgold ID: 111995), Sheffield, England, UK

Thank you for the opportunity to review this interesting article. Most of the comments provided are stylistic or requesting additional clarity, however there are some suggestions to enhance the discussion and recommendations of the work. 

**Background:**

 Para 2, 1 ^st^ sentence: “ *There is little evidence investigating”* should be – *“there is little research investigating”/”there is little evidence to show the effectiveness…”*

 Para 2, 2 ^nd^ sentence: *“One systematic review suggested the positive effect…”* – suggest ‘the’ replaced with ‘a’

 Para 2, 5 ^th^ sentence: *“effectively treat PLwD”* – should this be ‘care for’?

 Para 2, final sentence: *“It also sought to understand the thoughts and attitudes of support workers toward dementia and whether there is any correlation between knowledge and attitudes toward people working with dementia.”* – I think this must be an error – should it be “ living with dementia”?

**Methods:**

 Patient and public involvement – sentence 1 is rather anecdotal. Was this specific PPIE engagement concerned with this project, or was it drawn from literature (if so, cite it)?

 Paragraph 2 – JISC online surveys – needs a TM mark or company link. This is provided later in the section.

 Can you specify that higher scores for measures indicate higher levels of knowledge and more positive attitudes for the DKAS and DAS respectively? If that is in fact what higher scores indicate.

 Recruitment and consent – paragraph 1 - It isn't clear if this is just AHP support workers that you targeted or if it also includes nursing. If so, how did you publicise to nursing support workers?

 Data Collection – para 1 – *‘used by research team previously’* – is that relevant? 

 Data Collection - para 1 - *“Their data were included in the analysis” *– this would be better coming later, after you say that no revisions were needed.

 Data Collection, p5 top paragraph - *“All questions were optional to allow respondents to feel free to answer the questions they felt comfortable with.”* – What was the rationale for this? This topic is arguably not a highly sensitive topic and this was probably unnecessary and may have led to incomplete information. Unless all questions were answered and none missed, I would add this as a limitation.

 Data collection – final paragraph - This is a style issue, but I suggest this can easily be described in third person (e.g. The DKAS was used…), rather than ‘we/our’.

**Analysis:**

 Para 2 - *“For the validated questionnaires, we compared continuous data with categorical relationships (e.g mean DKAS/DAS scores vs. years of experience etc) parametric tests such as the Analysis of variance (ANOVA) were undertaken.”* – is there a word missing here? Please reword for clarity.

**Results:**

 Table 1 - The inclusion of a relatively high proportion (11%) of band 5/6 support workers is concerning.  It is extremely unusual to have support workers in bands 5 and 6 positions – does this mean they are no longer working as support workers? If so, I think these participants should be excluded from the sample as their views and knowledge as support workers are likely to now be influenced by their more senior positions.  

 P7 Years of experience and attitude towards people with dementia – final paragraph - *“These results imply that the length of time an individual has worked within the NHS does not significantly influence their attitudes towards dementia as measured by the DAS score.” *– not necessarily, suggest that the attitudes of those who remain in the role do not change over time. Attitudes may change in some individuals which may then result in them leaving their roles.

 P8 Occupational grade and knowledge of dementia - final sentence. *“This suggests that workers in higher occupational grades may possess greater knowledge about dementia.” *As the correlation between knowledge and occupational grade was weak and statistically non-significant, I would argue that the significant difference in knowledge scores between band 2 and 4 does not suggest that higher bands have more knowledge, but rather that those in band 2 are in starter positions and less likely to have increased knowledge of dementia due to a shorter period of experience. This is also mentioned in the discussion. Also, why were differences in knowledge level not examined between the other bandings? If there is a reason for this please provide it, if not, please complete the appropriate analysis.

 P8 Knowledge of dementia and attitude towards dementia - *“The Pearson correlation coefficient between DAS and DKAT scores was found to be approximately 0.35 (p=<0.01)”* – I am concerned by the use of the word “approximately”? If this means you have rounded the figure to two decimal places, I would remove the word “approximately”.

**Discussion:**

 General comment – This section does not appear to have fully engaged with the published literature. I am surprised that if the setting is the NHS that the authors haven't referenced any existing training resources, e.g. a quick search yielded a Dementia Training Standards Framework https://www.hee.nhs.uk/our-work/dementia-awareness/core-skills and further information here: https://www.hee.nhs.uk/our-work/dementia  What training is already 'out there' in the NHS (or charities), and what might be recommended? You say that support workers need 'more training' - we can always do with more training but what would be effective (and cost-effective)?

 The final sentence of the discussion appropriately discusses the importance of training which highlights abilities and potential of PLwD, but as mentioned above I am left wondering if these courses are already ‘out there’.  Are there existing training programmes that do this, and would it be feasible for the support workforce to tap into these (or are they too costly or too much time away from the ward)? Alternatively, do we need to create specific training for support workers? I think we need to see some more detail to be able to have a stronger take away message from this work.

 Final paragraph – *“Our data suggested a correlation between knowledge and attitude toward PLwD.”*  You noted earlier that this was a moderate positive correlation, so this should be added to this sentence.

**Conclusion**

 Final paragraph – *“a need for more comprehensive and specialised training programs” *– I think the message to readers / professions / policy makers could be stronger. Are the programs already available, whose responsibility is it to produce/deliver them, who should fund them, might they be online, in person, simulations? How can we deliver this training if support workers have such a small amount of the NHS training budget yet are one of the biggest groups of staff?

**Data availability**

 I prefer to see the survey questions (or a precis of them) housed within the article as these are essential to understanding the rigour and results of the study, e.g. as an appendix. However, that may depend on journal style and preferences.

Is the work clearly and accurately presented and does it cite the current literature?

Partly

If applicable, is the statistical analysis and its interpretation appropriate?

Yes

Are all the source data underlying the results available to ensure full reproducibility?

Yes

Is the study design appropriate and is the work technically sound?

Yes

Are the conclusions drawn adequately supported by the results?

Partly

Are sufficient details of methods and analysis provided to allow replication by others?

Yes

Reviewer Expertise:

Reviewer 1- Workforce research, support worker research; Reviewer 2 - support worker research, quantitative analysis

We confirm that we have read this submission and believe that we have an appropriate level of expertise to confirm that it is of an acceptable scientific standard, however we have significant reservations, as outlined above.

## References

[1] : Support workers knowledge, skills and education relating to dementia – a national survey. *NIHR Open Research* .2024;4: 10.3310/nihropenres.13671.1 10.3310/nihropenres.13671.1 39619687 PMC11605170

# Response

**Published:** 2024-11-25

**Authors:** Abi Hall

**Affiliations:** University of Exeter Medical School, Exeter, England, UK

The PPIE involvement  has been clarified in the text “For this study, specific patient and public involvement (PPIE) engagement was conducted to gather insights from individuals with lived experience of dementia care. Feedback from these sessions indicated that many patients and families felt clinicians often lacked a strong understanding of dementia and effective communication strategies. This input directly informed our study design and the survey content, helping us focus on areas where support workers might need additional training and resources.”

 TM/company link for JISC has been added

 We have added detail to explain that higher scores correlate to higher knowledge and more positive attitudes.

 Additional text has been added to clarify about recruitment “A targeted social media campaign using “X” (formerly Twitter) was used to advertise the survey, specifically aiming to reach Allied Health Professional (AHP) support workers and nursing support workers. Relevant professional bodies were contacted and asked to disseminate the survey link to their associate (non-registered) members, including those in nursing roles. Additionally, an email was sent to regional Allied Health Profession leads, who were asked to share the survey with both AHP and nursing support worker networks within their regions and organisations.”

 We have removed reference to this

 We have changed this

 Having all questions being optional has been added as a limitation

 This has been edited

 This has been reworded

 Some text has been added to the methods to clarify the inclusion of band 5/6 support workers – “Participants self-identified as support workers within the NHS, including those in Bands 2 to 6. Although Band 5 and 6 roles typically carry more senior responsibilities, we included these individuals as their primary role was reported as a support worker. This inclusion allows for a broader understanding of the knowledge and attitudes across the spectrum of support workers, although it may reflect a wider scope of experience.”

 And also to the limitations section “A small proportion (11%) of participants identified as Band 5 or Band 6, levels which generally entail more senior responsibilities beyond traditional support worker duties. While these participants identified as support workers, it is possible their views and knowledge could be influenced by higher-band responsibilities, which may differ from those in lower-band roles. Future studies might consider eligibility criteria to focus on Bands 2–4, ensuring the sample aligns closely with typical support worker roles.”

 While it is unusual, there are examples of support workers who do work at these banding and therefore, we would not wish to exclude them from the analysis, rather we have made reference to it in the text as a possible limitation

 We have added some additional text to clarify information about years of experience and attitudes towards dementia “These results suggest that, among those who remain in support worker roles, the length of time working within the NHS does not significantly influence attitudes toward dementia as measured by the DAS score. However, it is also possible that changes in attitudes could influence career trajectory, with some individuals who develop different perspectives or attitudes over time potentially leaving support worker roles. This potential attrition effect may mean that our sample reflects the attitudes of those who remain in the role, rather than capturing attitude changes over time within the broader population.”

 We have revised the occupational grade and knowledge section to highlight the weak correlation “The analysis revealed a significant difference in DKAS scores between Band 2 and Band 4 workers, with Band 4 workers scoring higher on average (mean DKAS score for Band 2 = 34.92, 95% CI: 32.46–37.37; mean DKAS score for Band 4 = 39.18, 95% CI: 37.11–41.25; t = -2.72, p < 0.01). However, given the weak, statistically non-significant overall correlation between occupational grade and dementia knowledge (Pearson correlation coefficient = 0.076, p = 0.452), this finding may reflect the relatively early career stage of Band 2 workers, who likely have less experience and fewer training opportunities specific to dementia. This difference, therefore, may not suggest that higher grades inherently possess greater dementia knowledge but rather that those in Band 2 are often in starter roles where dementia-specific knowledge is less developed.

 We chose to compare Bands 2 and 4 due to the clear delineation in occupational responsibilities and training levels typical of these grades. However, further analysis between other bands will be conducted to explore any additional differences in knowledge levels across occupational grades.

 “

 Addiditonal reference to relevant literature has been added in the next version.

 Additional text has been added to our discussion section “While our findings indicate gaps in knowledge and confidence, it is possible that some support workers may not have full access to or awareness of the DTSF resources. Therefore, improving accessibility and awareness of these established training pathways may be a cost-effective strategy for enhancing knowledge across support worker roles. Additionally, given that the DTSF and similar training frameworks are designed to provide standardised skills across different care roles, targeted reinforcement of the DTSF competencies could be beneficial, especially if integrated into routine supervision or mentorship programs within the NHS.

 To ensure training is both effective and cost-effective, future initiatives might consider integrating simulation-based training and peer support, which have been shown to improve practical skills in dementia care (Eggenberger et al., 2013). Simulation exercises could help support workers experience the challenges faced by individuals with dementia, fostering empathy and improving their ability to respond to challenging behaviours. Peer-led reflective sessions, where workers share experiences and discuss strategies for managing symptoms, could also support practical application of the DTSF competencies without requiring extensive additional resources.”

 This has been added

 We have revised the conclusion to add additional detail

 Thank you for your suggestion regarding the inclusion of survey questions. We agree that access to the survey items is important for understanding the study’s rigor and results. Due to space constraints and to maintain the readability of the main article, we have opted not to include the full survey within the text. Instead, we propose making the survey questions available as supplementary material, or as part of an online appendix, depending on journal style and guidelines. This approach would ensure that interested readers can review the questions in full without compromising the flow and structure of the article.

# Reviewer report: Reviewer response for version 1

**DOI:** 10.3310/nihropenres.14844.r32973

**Published:** 2024-11-08

**Authors:** Jan Oyebode

**Affiliations:** 1 University of Bradford, Bradford, England, UK

This paper reports the findings of an online survey of dementia support workers, asking about challenges faced in dementia care, knowledge, attitudes and dementia training. The survey was open for two months and recruited 109 participants. The findings showed quite high levels of knowledge and quite positive attitudes on standardized scales. Most staff had received in-house dementia training. The study revealed that key challenges were managing behavior that challenges and communication issues. There was a moderate correlation between attitudes and knowledge; no significant relationship between years of experience and knowledge or attitudes; but dementia knowledge was higher for those in grade 4 posts compared with those in grade 2 posts. Conclusions are drawn about the need for more training in communication and in behavior management.

 The report is very clearly written, well expressed, easy to follow and addresses an important topic . I would prefer the authors to avoid reference to the 'global epidemic' as this term is seen as reinforcing fears and stigma around dementia. The article is generally well situated in the context of the wider literature, although team and organizational levels of context were not addressed. So, for example, the study assumes that training is what provides knowledge and enhances attitudes but actually ongoing support, supervision and organizational culture may be important factors in enabling support staff to know how to respond to challenging behavior or how to communicate, over and above any formal training. I would like to see this acknowledged and see some recommendations or implications that address solutions other than training to address staff needs. A further omission is mention of the Dementia Training Standards Framework. All staff should be completing level 1 and 2 modules of this. This could be mentioned in the introduction or discussion, in relation to any recommendations around dementia training

 The data collection was well designed and was piloted. I think the survey was limited to England rather than being open across the UK. This may need stating more plainly. Online surveys may not be readily accessible to some groups, including those with limited resources, limited literacy or English fluency. These are all characteristics which may apply to some support workers. It is notable that the respondents were mainly of white ethnicity and this could reflect that limitation. This could be acknowledged in the limitations section with recommendations for how to address these 'EDI' issues in future research.

 The relatively small number of staff responding was probably affected by timelines, as the survey was only open for 8 weeks. However, there were sufficient respondents to conduct descriptive and  correlational statistics.

 The findings section is well structured. I did not understand the statement that the “occupational grade” variable was numerically coded, as the comparison was between grades 2 and 4.

 I would have liked to see some additional brief information about the basic levels of knowledge and nature of attitudes in the sample. I think the knowledge levels exceed those found in support workers in the standardization studies, which could imply the training in the UK has had a good effect.

 There is a crucial typographical error in table 1 where it says that only 3 respondents were at grade 4 - I think it should be 30 something?

 In the discussion there is an assumption that the correlation between knowledge and attitudes shows training leads to knowledge which leads to positive attitudes. This should be softened, given correlations are only associations and that also we do not know that training was responsible for the knowledge.

 Overall, this is a good article and only minor changes to intro and discussion are needed to enhance its content.

Is the work clearly and accurately presented and does it cite the current literature?

Partly

If applicable, is the statistical analysis and its interpretation appropriate?

Yes

Are all the source data underlying the results available to ensure full reproducibility?

Yes

Is the study design appropriate and is the work technically sound?

Yes

Are the conclusions drawn adequately supported by the results?

Partly

Are sufficient details of methods and analysis provided to allow replication by others?

Yes

Reviewer Expertise:

Dementia care research including young onset dementia, ethnicity and dementia, dementia training and education.

I confirm that I have read this submission and believe that I have an appropriate level of expertise to confirm that it is of an acceptable scientific standard, however I have significant reservations, as outlined above.

# Response

**Published:** 2024-11-25

**Authors:** Abi Hall

**Affiliations:** University of Exeter Medical School, Exeter, England, UK

Thank you for your comprehensive review. We have made the following changes.

 We have removed reference to the global epidemic.

 We agree that team and organisational factors, including supervision, ongoing support, and a supportive organisational culture, play critical roles in enabling support staff to respond effectively to challenging behaviour and communicate effectively with individuals living with dementia. Although our study primarily focused on the role of formal training, we recognise that a holistic approach—encompassing these additional elements—may have a more profound impact on staff preparedness and resilience.

 Additional text has been added to the discussion

 “While our study highlights the role of training in enhancing dementia knowledge and improving attitudes among support workers, we recognise that training alone may not be sufficient to prepare staff for the complexities of dementia care. Existing literature suggests that a supportive organisational environment, regular supervision, and access to mentorship are essential in reinforcing knowledge and sustaining positive attitudes. Organisational culture that fosters openness, empathy, and collaboration may empower support workers to address challenging behaviours and communication barriers in ways that training alone cannot achieve. Ongoing support from supervisors and peers, along with structured opportunities for reflection and feedback, can be especially effective in helping staff apply learned skills in real-world contexts. This layered approach, integrating formal training with organisational and team-based support, is likely to build resilience and confidence, ultimately improving the quality of dementia care.”

 Text has been added to the introduction referencing the DTSF

 “The Dementia Training Standards Framework (DTSF) , provides a structured guide for dementia training across health and social care sectors. The framework outlines key competencies at various levels, recommending that all staff, including support workers, complete level 1 (awareness) and level 2 (intermediate) training to ensure a foundational understanding of dementia care. Although the DTSF is widely recognised for setting baseline competencies in dementia care across health and social care roles, but specific evaluations of its impact on support workers, especially in terms of long-term outcomes, are limited.”

 We reference the fact that the survey was undertaken for support workers in England in several places in the abstract and methods sections.

 The following text has been added to the limitations section “Our study relied on an online survey platform, which may not have been readily accessible to all support workers. Barriers such as limited digital resources, literacy challenges, and English fluency could have restricted participation among certain groups, potentially skewing the demographics of our sample. Notably, the majority of respondents identified as white British, which may reflect an accessibility limitation that could affect the applicability of our findings.”

 Unfortunately we were under significant time limitations for this work so the survey was only open for a short period of time.

 Further clarification and explanation has been added – “For the purpose of statistical analysis, the "occupational grade" variable was numerically coded to enable comparisons. Each NHS band (e.g., Bands 2, 3, 4) was assigned a corresponding numeric value to facilitate statistical testing. For example, comparisons were made between Bands 2 and 4 by using these coded values to compute t-tests and correlations, allowing us to examine potential differences in knowledge and attitudes between these groups.”

 We have added some detail  about the basic levels of knowledge and attitudes in the sample. “The knowledge levels observed in our sample, as measured by the DKAS, appear higher than those reported in previous standardisation studies among support workers . This may suggest that dementia training programs in England, guided by frameworks like the DTSF, have a positive effect on knowledge levels. Similarly, the attitudes observed in our sample align with positive attitudes reported by O’Connor and McFadden  in their work with the Dementia Attitudes Scale (DAS), indicating that training may also support compassionate attitudes in dementia care settings.”

 We have checked our data and we did only have 3 respondents who reported they were Band 4

 We have edited the text to reflect this the assumption that there is a correlation between knowledge and attitudes. “Our data suggested a positive correlation between knowledge and attitudes toward people living with dementia (PLwD). However, it is essential to note that correlation does not imply causation, and the association should not be interpreted as direct evidence that increased knowledge causes improved attitudes. While training may contribute to the knowledge levels observed, our study did not assess the specific sources of knowledge, meaning we cannot confirm that training alone is responsible. Other factors, such as previous experience with PLwD or supportive organisational culture, may also influence knowledge and attitudes. Future research could explore these factors further, using longitudinal designs to assess how training, experience, and support mechanisms interact to shape both knowledge and attitudes.”
